# Sex of the Treating Rheumatologist and Disease Activity Outcomes in Psoriatic Arthritis

**DOI:** 10.1016/j.mayocpiqo.2026.100761

**Published:** 2026-09-22

**Authors:** Alessandro Giollo, Mariangela Salvato, Kiren Khalid, Lorenzo Di Luozzo, Riccardo D’Andrea, Roberta Ramonda

**Affiliations:** a Rheumatology Unit, Department of Medicine - DIMED, University of Padua, Italy; b Rheumatology Unit, Department of Medicine, University Hospital of Padua, Italy

## Abstract

**Objective:**

To assess whether the sex of the treating rheumatologist is associated with longitudinal disease activity trajectories in patients with psoriatic arthritis (PsA).

**Patients and Methods:**

We conducted a longitudinal registry-based study including 712 patients with PsA followed from 26 September 2014 to 23 December 2019 and classified by rheumatologist sex at baseline. The primary outcome was change from baseline in 28-joint disease activity score based on C-reactive protein (DAS28-CRP) (n=588) and ankylosing spondylitis disease activity score based on C-reactive protein was secondary (n=452). Missing baseline covariates were multiply imputed. Baseline imbalances were addressed using inverse probability of treatment weighting based on outcome-specific propensity scores. Longitudinal associations were estimated using inverse probability of treatment weighting-weighted generalized estimating equations with spline modeling of time and pooled using Rubin’s rules.

**Results:**

Patients (410/712 [57.6%] managed by male and 302/712 [42.4%] by female rheumatologists) had a median follow-up of 3.4 years. At 12 months, the pooled difference in ΔDAS28-CRP (women-men) was -0.08 (95% CI, −0.37 to 0.20; *P*=.577). Differences were larger at 24 months (−0.382; 95% CI, −0.62 to −0.14; *P*=.002) than at 12 months, whereas the 36-month estimate remained directionally similar (−0.26; 95% CI, −0.54 to 0.01; *P*=.061). The 24-month difference coincided with larger reductions in 28-joint tender joint count (−0.85; 95% CI, −1.53 to −0.17) and 28-joint swollen joint count (−0.62; 95% CI, −1.18 to −0.05). No between-group differences were observed for ankylosing spondylitis disease activity score based on C-reactive protein. Estimates were generally similar across sensitivity analyses.

**Conclusion:**

In this real-world PsA cohort, rheumatologist sex was associated with a modestly greater mean DAS28-CRP improvement at 24 months, mainly driven by joint counts.

Psoriatic arthritis (PsA) is a chronic immune-mediated inflammatory disease associated with pain, functional impairment, and reduced quality of life. Over the past 2 decades, earlier diagnosis and the expansion of biologic and targeted-synthetic disease-modifying antirheumatic drugs (b/tsDMARDs) have enabled treat-to-target approaches, yet a substantial proportion of patients do not achieve sustained minimal disease activity or remission in real-world care.^1^, ^2^, ^3^ Although multiple patient-level determinants of treatment response and disease burden have been investigated, less is known about the impact of physician-level factors on long-term outcomes in PsA.

In other medical specialties, the sex of the treating physician has been associated with differences in processes of care and patient outcomes. Large observational analyses have reported lower mortality and improved outcomes among patients treated by female physicians in general medicine and across common surgical procedures, suggesting that physician-related characteristics may be associated with clinically meaningful differences in care delivery and outcomes.^4^^,^^5^ Mechanistic explanations have focused on differences in communication style and patient-centeredness; meta-analytic evidence indicates that the sex of the treating physician can influence medical communication patterns,^6^ and observational work has highlighted variations in care processes between female physicians and male physicians.^7^ In addition, the sex of the treating physician has been discussed as a potential source of implicit bias that may affect clinical decision-making,^8^ thus reinforcing the importance of assessing physician-related determinants of outcomes.

In rheumatic and musculoskeletal diseases, disease assessment and treatment decisions rely heavily on composite indices integrating physician-reported and patient-reported measures, making the clinical encounter itself a potential determinant of outcomes. Several studies in PsA and related conditions have documented substantial discordance between patient and physician global assessments, with female sex, pain burden, psychological distress, and educational level emerging as independent contributors to divergent disease perceptions.^9^^,^^10^ Mental health status has been consistently associated with overestimation of disease severity by patients compared with physicians in psoriasis and PsA, underscoring the complex interplay between psychological factors and disease evaluation.^9^^,^^11^ Beyond patient-level determinants, physician-related factors such as communication style and shared decision-making approaches have been shown to influence the quality of the patient–doctor relationship and treatment processes in rheumatic and musculoskeletal diseases.^12^ Recent evidence further indicates that physicians’ tolerance of uncertainty varies with age and practice experience and is related to shared decision-making perceptions and practices.^13^ These data support treating physician sex as a marker of potentially modifiable professional behaviors and organizational processes, rather than as a mechanistic or biological exposure.

Emerging prospective evidence in rheumatoid arthritis (RA) has challenged the assumption that the sex of the treating physician matters only for relational outcomes. In a long-term cohort of patients with RA treated with b/tsDMARDs at our center, management by female rheumatologists was associated with lower disease activity and higher remission odds than management by male rheumatologists, independent of patient characteristics and physician experience.^14^ This finding provides a strong rationale to examine whether similar associations exist in PsA, in which outcome measurements, treatment targets, and physician-patient shared decision-making may differ from RA.

## Patients and Methods

### Study Population and Design

We analyzed longitudinal data from the single-center, encounter-based electronic rheumatology registry of the Rheumatology Unit, University Hospital of Padua. Since 2014, it has been mandatory for every physician at our center to record all candidates for b/tsDMARD treatment in a forward-looking electronic registry known as the Piattaforma Servizi Farmaceutici. The registry may only be completed by licensed physicians who have the authority to prescribe b/tsDMARDs. Consequently, the registry of patients who received b/tsDMARD prescriptions from our department and were monitored by our personnel should be considered comprehensive. Outcomes, exposures, and effect modifiers were gathered within the Piattaforma Servizi Farmaceutici. The registry prospectively captures clinical assessments, patient-reported measures, laboratory results and treatments recorded during routine outpatient visits; visit timing and frequency therefore reflect clinical practice rather than a protocol-mandated schedule.

As patients entered during routine follow-up, this was a prevalent cohort. Repeated assessments after each patient's baseline were included until the last available follow-up.

The study was approved by the Central-Eastern Veneto Area Territorial Ethics Committee with protocol number 6007/AO/24 in compliance with the principles of the Declaration of Helsinki as revised in 2000. The study is reported in accordance with the Strengthening the Reporting of Observational Studies in Epidemiology statement; the completed Strengthening the Reporting of Observational Studies in Epidemiology checklist is provided as a separate supplemental file.

### Inclusion and Exclusion Criteria

Eligible participants were adults (>18 years) with a clinical diagnosis of PsA, at least one eligible registry visit from 26 September 2014 to 23 December 2019, and an identifiable treating rheumatologist. We did not implement any formal exclusion criteria.

### Outcomes

At each recorded visit, peripheral disease activity was assessed with the disease activity score in 28 joints (DAS28-CRP), the primary outcome. DAS28-CRP integrates 28-joint tender count (TJC28), 28-joint swollen count (SJC28), patient global assessment (PGA) of disease activity, and C-reactive protein (CRP); higher values indicate greater disease activity. Longitudinal analyses modeled change from the individual baseline, with negative values indicating improvement. Clinically important DAS28-CRP improvement was defined as a decrease of at least 0.6, with a decrease of at least 1.2 examined as a larger response. Ankylosing spondylitis disease activity score based on C-reactive protein (ASDAS-CRP), which integrates back pain, duration of morning stiffness, peripheral joint pain/swelling, PGA, and CRP, was analyzed as an exploratory secondary outcome; decreases of at least 0.6 (exploratory) and 1.1 were used as response thresholds.

Because axial involvement was not formally recorded, ASDAS-CRP was available for a subset of the overall cohort and was not restricted to patients with confirmed axial PsA. It therefore cannot be interpreted as a validated axial-domain analysis in this dataset. Individual components of both composite indices were also analyzed.

### Covariates

The following variables were prospectively collected within the registry: disease duration in years, weight, height, TJC28, SJC28, CRP (in mg/L), PGA (0-100), morning stiffness (0-10; Bath Ankylosing Spondylitis Disease Activity Index (BASDAI) question : “How long does your morning stiffness last from the time you wake up?”), back pain (0-10; BASDAI question: “How would you describe the overall level of AS neck, back, or hip pain you have had?”), peripheral joint pain/swelling (0-10; BASDAI question: “How would you describe the overall level of pain/swelling in joints other than neck, back, or hips you have had?”), comorbidities (yes/no), extra-articular involvement (yes/no; referring to uveitis, IBD, psoriasis, enthesitis, or dactylitis), erosive disease (yes/no), radiographic progression within 12 months (yes/no), prior DMARDs, current prescription of b/tsDMARD, current prescription of regular NSAID therapy (yes/no), glucocorticoid dose (in mg equivalent of prednisone), and the health assessment questionnaire (HAQ).

### Exposure

The primary exposure was the sex of the treating rheumatologist at each patient’s first eligible registry visit. This baseline classification was retained for the primary analysis to preserve temporal ordering. Because patients could subsequently be seen by different clinicians, complementary longitudinal exposure analyses used visit-updated rheumatologist sex, restriction to patients who never changed rheumatologist sex, stratification by whether a change occurred, and classification according to the sex, accounting for at least 50% of follow-up visits.

### Missing Data Handling

Patients required an observed baseline value of the outcome used to construct a change score; accordingly, the DAS28-CRP and ASDAS-CRP analytic cohorts included 588 and 452 patients, respectively. Intermittently missing follow-up outcomes were not imputed. Missingness by exposure group is reported in Supplemental Table 1 (available online at http://www.mcpiqojournal.org). Baseline covariates were multiply imputed by fully conditional specification, generating 50 datasets. Continuous and count variables used predictive mean matching based on Bayesian linear predictions, and binary variables used Bayesian logistic models. Imputation models included the exposure, all propensity-score covariates, calendar year, variable-level missingness indicators, and longitudinal auxiliary measures; DAS28-CRP and ASDAS-CRP were passively recalculated from their imputed components. Estimates and their variances were combined with Rubin’s rules. The analysis assumes that missing covariate values are missing at random conditional on the imputation model.

### Propensity-Score Weighting

Within each imputed dataset, a logistic propensity-score (PS) model estimated the probability of baseline management by a female rheumatologist. The PS was estimated separately in the DAS28-CRP (n=588) and ASDAS-CRP (n=452) analytic cohorts so that balance targeted the population entering each outcome analysis. Prespecified covariates were age, patient sex, disease duration, body mass index, obesity, comorbidities, extra-articular manifestations, DAS28-CRP, ASDAS-CRP, TJC28, SJC28, PGA, HAQ, CRP, erosions, radiographic progression during the preceding 12 months, NSAID use, glucocorticoid dose, and previous b/tsDMARD exposure. Stabilized inverse probability weights targeting the average treatment effect were truncated at the within-imputation 1st and 99th percentiles. Positivity and influential weights were assessed graphically and by weight range and effective sample size (Supplemental Figure 1, available online at http://www.mcpiqojournal.org). Balance was summarized within each imputation using absolute standardized mean differences (SMDs) and then averaged across imputations; <0.10 was the prespecified diagnostic threshold. For incomplete covariates, observed-value and missingness indicator SMDs were also shown separately as diagnostics of differential ascertainment (Supplemental Table 2, available online at http://www.mcpiqojournal.org).

### Statistical Analyses

#### Descriptive analysis

Continuous variables are reported as median (IQR), and categorical variables as number/denominator (%). Unweighted comparisons used the Mann–Whitney U test and Pearson χ^2^ or Fisher exact test, as appropriate.

#### Primary longitudinal analysis

Longitudinal changes in disease activity (DAS28 and ASDAS) were analyzed using inverse probability of treatment weighting (IPTW)-weighted generalized estimating equations (GEE), which estimate population-averaged effects while accounting for within-patient correlation of repeated measurements. GEE models were preferred over subject-specific mixed-effects models because the primary objective was to estimate average population-level differences in disease activity trajectories according to rheumatologist sex, rather than individual level predictions. Outcomes were modeled as change from baseline (ΔDAS28, ΔASDAS) to facilitate interpretation of longitudinal differences and to account for baseline heterogeneity in disease activity between exposure groups. Baseline disease activity was therefore not included as an additional covariate in the outcome models to avoid redundancy and collinearity. Weighted GEE models were fitted without further covariate adjustment, relying on IPTW to account for baseline confounding. An exchangeable working correlation structure was specified as the primary assumption. Robust sandwich variance estimators were used to ensure valid inference even in the presence of misspecification of the correlation structure. Time since baseline was modeled using restricted cubic splines to allow for nonlinear disease activity trajectories over follow-up, without imposing parametric assumptions. Four degrees of freedom were selected a priori to balance flexibility and parsimony. An interaction term between time and rheumatologist sex was included to allow differential longitudinal trajectories between exposure groups. Effect estimates at specific follow-up times were derived from the fitted spline models and reflect differences in adjusted mean change from baseline at the corresponding time points. In addition to the composite DAS28-CRP and ASDAS-CRP, their individual components (TJC28, SJC28, PGA, CRP, back pain, morning stiffness, and joint pain/swelling) were analyzed separately using identical IPTW-weighted GEE specifications.

#### Sensitivity analyses

Robustness of the DAS28-CRP effect estimates at 12, 24, and 36 months was evaluated using nontruncated stabilized weights, an autoregressive working correlation structure and alternative spline specifications, a PS estimated in the full eligible cohort (n=712), and a PS excluding HAQ (Supplemental Table 3, available online at http://www.mcpiqojournal.org).

Because changes in treating rheumatologists could occur during follow-up, sensitivity analyses updated rheumatologist sex at each visit, restricted the cohort to patients with stable physician sex exposure, stratified patients according to whether physician sex changed, and classified patients by predominant longitudinal exposure (female rheumatologists at ≥50% of visits). For baseline, stable, changed, and predominant-exposure analyses, the visit closest to 12, 24, and 36 months within a ±6-month window was selected; IPTW-weighted between-group differences and robust 95% CI were estimated within each imputed dataset and pooled using Rubin’s rules.

A further post hoc sensitivity analysis was undertaken in response to the residual balance diagnostics. The primary truncated-weight, exchangeable GEE was additionally adjusted for every baseline variable with a mean post-weighting absolute SMD ≥0.10 for either its observed values or its missingness indicator. These variables were disease duration, ASDAS-CRP, HAQ, and baseline-recorded radiographic progression during the preceding 12 months. Within each imputed dataset, the multiply imputed values of these covariates were used, with continuous covariates subsequently standardized. A separate missingness indicator was included for each selected covariate (Supplemental Table 4, available online at http://www.mcpiqojournal.org).

#### Subgroup analyses

Effect modification by patient sex and age (<65 vs ≥65 years) was explored using the corresponding interaction terms. Using the visit closest to 12 months within the same ±6-month window, IPTW-weighted risks and risk ratios were estimated for clinically important improvement in DAS28-CRP (decrease ≥0.6 and ≥1.2) and ASDAS-CRP (decrease ≥0.6 and ≥1.1).

Physician case volume was defined as the number of distinct study patients attributed to each rheumatologist over the complete observation period and was dichotomized at the physician-level median. This variable represents study-period caseload rather than annual workload. Effect modification of the association with a DAS28-CRP decrease ≥0.6 at 12 months by physician case volume was assessed on the log-risk-ratio scale.

#### Changes in b/tsDMARDs

Changes in b/tsDMARD therapy during follow-up, including treatment switching, were considered post-baseline processes potentially influenced by the treating rheumatologist. Any treatment switch, exactly one switch, and multiple switches were examined as secondary post-baseline outcomes using full-cohort IPTW-weighted risks and risk ratios (RRs).

No multiplicity adjustment was applied to secondary, component, subgroup, or sensitivity analyses; these results were therefore considered exploratory and interpreted primarily according to effect magnitude and precision.

Effect estimates are presented with two-sided 95% CIs. A two-sided *P*<.05 was used for the prespecified 12-month primary contrast. Retrospective observed power was not used to interpret the findings. To minimize potential investigator-related bias, none of the authors involved in data extraction or statistical analyses were prescribing physicians in the study database. The senior author, who was a prescriber, was not involved in data handling or statistical modeling. Analyses were conducted using Python version 3.12.

## Results

### Study Population

The study flow and derivation of the outcome-specific analytic cohorts are shown in Figure 1. Among 712 PsA patients, 410 (57.6%) were managed by men and 302 (42.4%) by women rheumatologists (Table 1). The median follow-up duration was 3.4 years (IQR 1.4-4.5), with a median of 8 visits per patient (IQR 4-11). Sixteen rheumatologists (5 women and 11 men) contributed at baseline. At the physician-level, median baseline caseload was 11 patients (range 4-266) for female rheumatologists and 33 patients (range 1-102) for male rheumatologists; one female rheumatologist accounted for 266 of the 302 patients in the female rheumatologist group. The age of male rheumatologists and female rheumatologists was comparable (median 62 years, IQR 43-64.5 vs 61 years, IQR 60-61; *P*=.91).

**Figure 1:**
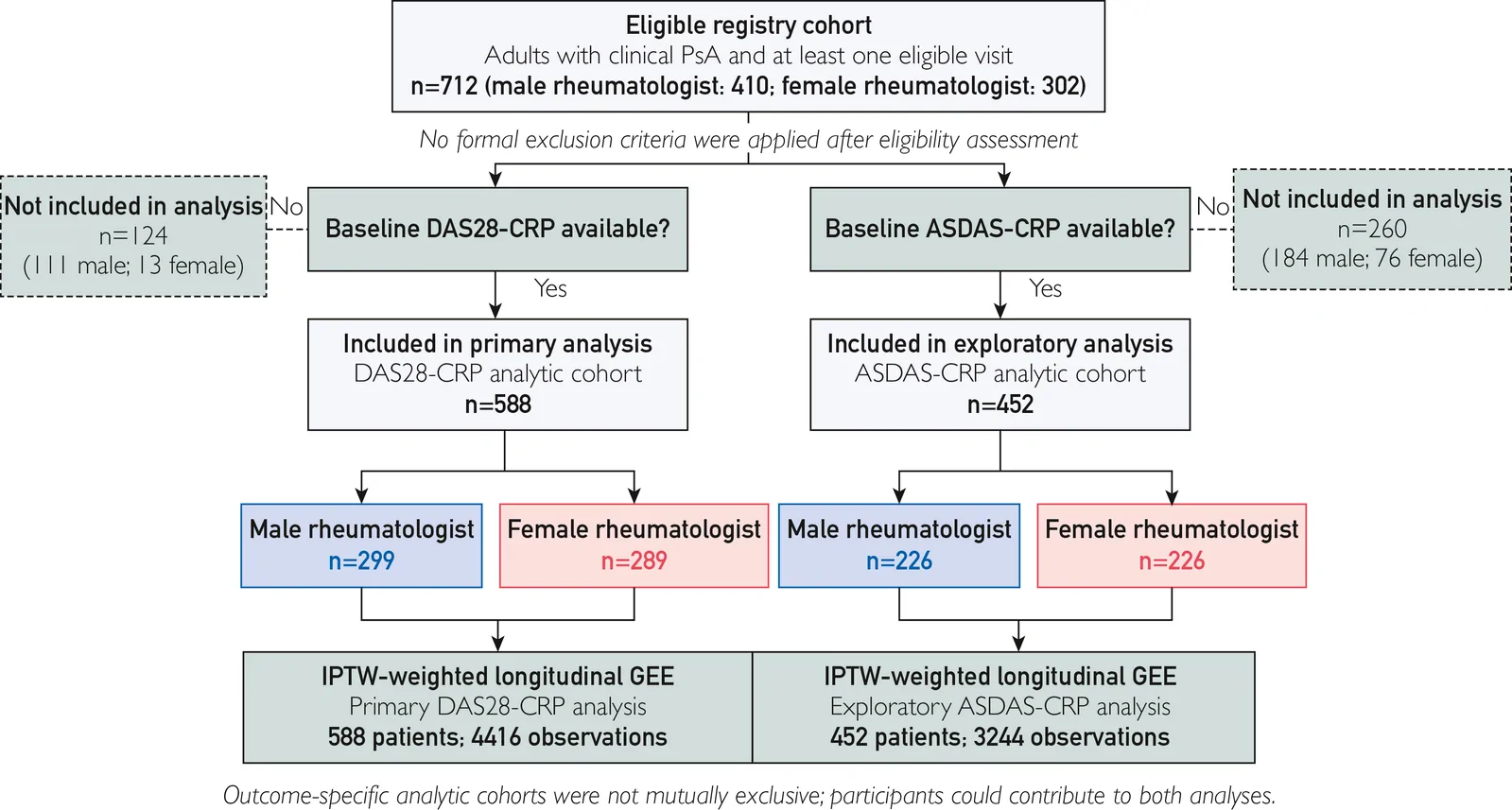
Study flow diagram and outcome-specific analytic cohorts. ASDAS-CRP, ankylosing spondylitis disease activity score based on C-reactive protein; DAS28-CRP, 28-joint disease activity score based on C-reactive protein; GEE, generalized estimating equations; IPTW, inverse probability of treatment weighting.

**Table 1:** Baseline Characteristics (Unweighted) of the Study Population, According to Rheumatologist’s Sex

Baseline characteristics	Male Rheumatologist (n=410)	Female Rheumatologist (n=302)	*P*
Patient’s characteristics			
Age, y	52.89 (46.26-60.85)	53.97 (44.38-60.67)	.84
Female patient, n (%)	200/410 (48.8%)	145/302 (48.0%)	.84
Disease duration, y	9.00 (4.00-15.00) n=404	11.00 (5.00-17.00) n=301	.014
BMI, kg/m^2^	25.95 (23.38-29.00)	24.81 (22.72-27.81)	.008
Obesity, n (%)	79/410 (19.3%)	43/302 (14.2%)	.078
Extra-articular manifestations, n (%)	159/410 (38.8%)	138/302 (45.7%)	.064
Comorbidities, n (%)	149/313 (47.6%)	82/226 (36.3%)	.009
Disease activity			
DAS28-CRP	3.98 (2.79-5.13) n=299	3.79 (2.66-4.51) n=289	.003
ASDAS-CRP	2.55 (1.98-3.49) n=226	2.88 (1.90-3.52) n=226	.58
SJC28	2.00 (0.00-6.00)	1.00 (0.00-4.00)	<.001
TJC28	5.00 (2.00-10.00)	4.00 (1.00-7.00)	<.001
PGA 0-100	50.00 (31.25-80.00)	50.00 (30.00-70.00)	.17
Back pain 0-10	4.00 (3.00-8.00)	5.00 (2.00-7.00)	.67
Peripheral pain/swelling 0-10	4.00 (2.00-7.00)	5.00 (2.00-7.00)	.57
Morning stiffness 0-10	4.00 (3.00-7.00)	5.00 (3.00-7.00)	.57
HAQ	1.25 (0.70-1.60) n=99	0.75 (0.25-1.30) n=29	.023
CRP, mg/L	3.00 (1.60-6.00)	4.00 (2.90-7.00)	.001
Erosions, n (%)	181/301 (60.1%)	161/289 (55.7%)	.28
Radiographic progression, n (%)	69/203 (34.0%)	81/251 (32.3%)	.70
Medications			
Prednisone dose, mg/d	0.00 (0.00-5.00)	0.00 (0.00-5.00)	.020
NSAID use, n (%)	182/409 (44.5%)	164/300 (54.7%)	.007
Current csDMARD use, n (%)	125/410 (30.5%)	62/302 (20.5%)	.003
Prior b/tsDMARD exposure, n (%)	168/410 (41.0%)	161/302 (53.3%)	.001

Abbreviations: ASDAS-CRP, ankylosing spondylitis disease activity score based on C-reactive protein; BMI, body mass index; b/tsDMARD, biologic or targeted-synthetic disease-modifying antirheumatic drug; BP, back pain; CRP, C-reactive protein; DAS28-CRP, 28-joint disease activity score based on C-reactive protein; HAQ, health assessment questionnaire; IL, interleukin; JP, joint pain; MS, morning stiffness; NSAID, non-steroidal anti-inflammatory drug; PGA, patient global assessment; SJC28, 28-joint swollen joint count; TJC28, 28-joint tender joint count.

Patients treated by female rheumatologists had longer disease duration and lower peripheral disease activity (DAS28-CRP, TJC28, and SJC28), despite marginally higher CRP levels. PGA, extra-articular manifestations, erosive disease, and radiographic progression were similar. They had fewer comorbidities and a slightly lower body mass index. Previous b/tsDMARD exposure and NSAID use were more frequent in the female rheumatologist group. A concomitant csDMARD was prescribed in 20.5% of the female rheumatologist group and 30.5% of the male rheumatologist group.

### Follow-up

Across the entire follow-up period, a total of 21 rheumatologists contributed to patient care, including 14 men and 7 women. During follow-up, 287 of 712 patients (40.3%) experienced at least one change in treating physician, and 175 patients (24.6%) experienced at least one change in the sex of the treating physician. Most patients had 1 or 2 changes, while repeated switches were uncommon; baseline exposure agreed with predominant follow-up exposure in 680/712 patients (95.5%).

### IPTW

Baseline DAS28-CRP was observed in 588 patients (299 male rheumatologist exposure and 289 female rheumatologist exposure) and ASDAS-CRP in 452 (226 in each group). HAQ was observed in 128/712 patients, and radiographic progression in 454/712 (Supplemental Table 1). In the primary DAS28-CRP cohort, the mean trimmed stabilized weight across 50 imputations was 0.987 (mean SD, 0.501), the mean truncation limits were 0.527 and 3.554, and the mean effective sample size was 467.7. All mean absolute post-weighting SMDs for MI-completed covariates were <0.10 (largest 0.057). Nevertheless, weighted SMDs among observed records remained 0.164 for ASDAS-CRP, 0.283 for HAQ, and 0.253 for radiographic progression; corresponding missingness SMDs were 0.548, 0.349, and 0.568, and disease duration missingness SMD was 0.128 (Supplemental Table 2).

### Longitudinal DAS28 Trajectories by the Sex of the Treating Physician

The primary DAS28-CRP analysis included 588 patients and 4416 observations (Figure 2; Table 2). At 12 months, the estimated difference in ΔDAS28-CRP (female-male rheumatologist) was −0.081 (95% CI, −0.367 to 0.205; *P*=.577). At 24 months, the difference favored female rheumatologists and was larger (−0.382; 95% CI, −0.621 to −0.143; *P*=.002). The 36-month estimate remained directionally similar (−0.262; 95% CI, −0.535 to 0.012; *P*=.061).

**Figure 2:**
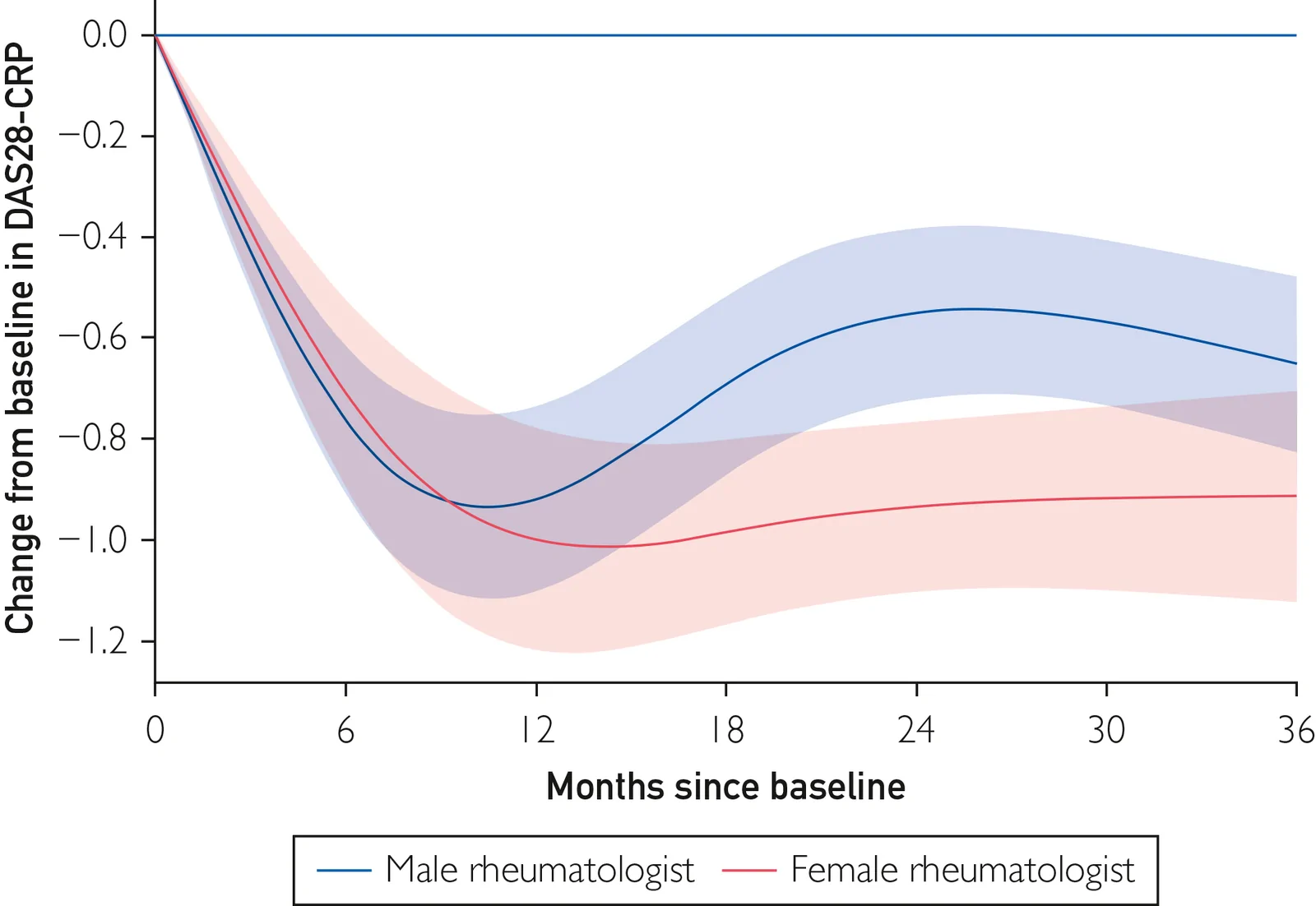
Predicted changes from baseline in DAS28-CRP by baseline rheumatologist sex. Curves and 95% confidence bands derive from outcome-specific stabilized IPTW-weighted GEE models fitted in 50 imputed datasets and combined with Rubin's rules. Time was modeled with restricted cubic splines (four degrees of freedom); predictions were centered at zero at baseline. DAS28-CRP, 28-joint disease activity score based on C-reactive protein; GEE, generalized estimating equations.

**Table 2:** Adjusted Changes in DAS28-CRP Over Time by Rheumatologist Sex Derived From IPTW-Weighted GEE Models, Including Estimates at 12, 24, and 36 Months

Endpoint	ΔDAS28 male rheumatologist	ΔDAS28 female rheumatologist	Female-male difference	95% CI	*P*
12-mo contrast	−0.916	−0.997	−0.081	−0.367 to 0.205	.58
24-mo contrast	−0.551	−0.933	−0.382	−0.621 to -0.143	.002
36-mo contrast	−0.651	−0.913	−0.262	−0.535 to 0.012	.061

Abbreviations: DAS28-CRP, 28-joint disease activity score based on C-reactive protein; GEE, generalized estimating equations; IPTW, inverse probability of treatment weighting.

Estimates were similar with untruncated weights, an autoregressive correlation, alternative spline flexibility, the full-cohort PS, and exclusion of HAQ from the PS; the 24-month contrast remained negative in every specification, whereas precision at 36 months varied (Supplemental Figure 2, available online at http://www.mcpiqojournal.org; Supplemental Table 3). Further adjustment for disease duration, ASDAS-CRP, HAQ, and radiographic progression plus their missingness indicators produced differences of −0.065 (95% CI, −0.346 to 0.216; *P*=.650), −0.371 (−0.608 to −0.135; *P*=.002), and −0.249 (−0.521 to 0.022; *P*=.072) at 12, 24, and 36 months, respectively (Supplemental Table 4).

At 24 months, exploratory component differences were −0.853 for TJC28 (95% CI, −1.533 to −0.173; *P*=.014) and −0.615 for SJC28 (−1.177 to −0.053; *P*=.032) (Figure 3; Table 3). Differences in PGA (−1.291; −6.581 to 3.999) and CRP (−0.045 mg/L; −1.563 to 1.472) included the null. At 12 and 36 months, all 4 component contrasts included the null. These component analyses were not multiplicity-adjusted.

**Figure 3:**
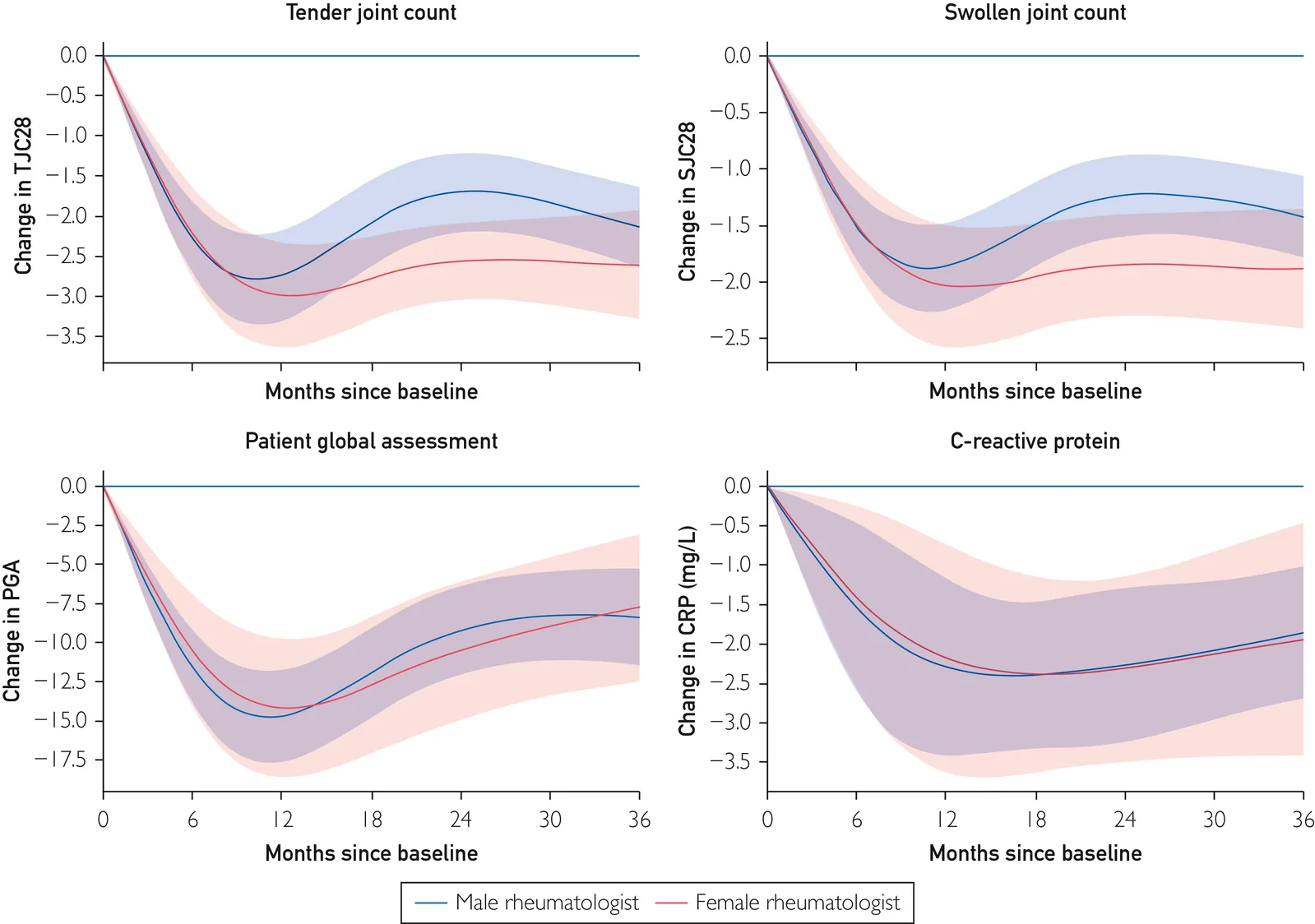
Predicted changes from baseline in DAS28-CRP components by baseline rheumatologist sex. Panels show TJC28, SJC28, PGA and CRP trajectories from identically specified IPTW-weighted GEE models. Component analyses were exploratory and not multiplicity-adjusted. CRP, C-reactive protein; DAS28-CRP, 28-joint disease activity score based on C-reactive protein; GEE, generalized estimating equations; IPTW, inverse probability of treatment weighting; PGA, patient global assessment; SJC28, 28-joint swollen joint count; TJC28, 28-joint tender joint count.

**Table 3:** Exploratory Pooled Female-Minus-Male Rheumatologist Differences in DAS28-CRP Component Changes

Outcome	12-Mo Difference (95% CI)	24-Mo Difference (95% CI)	36-Mo Difference (95% CI)
TJC28	−0.248 (−1.113 to 0.618)	−0.853 (−1.533 to -0.173)	−0.474 (−1.309 to 0.362)
SJC28	−0.177 (−0.838 to 0.484)	−0.615 (−1.177 to -0.053)	−0.467 (−1.098 to 0.165)
PGA	+0.486 (−4.825 to 5.796)	−1.291 (−6.581 to 3.999)	+0.619 (−5.084 to 6.322)
CRP, mg/L	+0.107 (−1.745 to 1.959)	−0.045 (−1.563 to 1.472)	−0.096 (−1.776 to 1.584)

Abbreviations: CRP, C-reactive protein; DAS28-CRP, 28-joint disease activity score based on C-reactive protein; PGA, patient global assessment; SJC28, 28-joint swollen joint count; TJC28, 28-joint tender joint count.

With visit-updated rheumatologist sex, DAS28-CRP differences were −0.050 (95% CI, −0.326 to 0.227; *P*=.724), −0.369 (−0.597 to −0.142; *P*=.001), and −0.317 (−0.573 to −0.061; *P*=.015) at 12, 24, and 36 months, respectively (Supplemental Figure 3; Supplemental Table 5, available online at http://www.mcpiqojournal.org).

Closest-window analyses yielded the same qualitative 12-month null and later negative pattern. Among stable-exposure patients, differences were −0.001 (95% CI, −0.357 to 0.356; n=371), −0.408 (−0.768 to −0.048; n=292), and −0.491 (−0.934 to −0.047; n=240) at 12, 24, and 36 months. Among patients whose physician sex exposure changed, estimates were −0.226 (−0.717 to 0.265; n=126), −0.423 (−1.010 to 0.163; n=114), and −0.626 (−1.211 to −0.041; n=98). Predominant-exposure estimates were −0.018, −0.381, and −0.474, respectively (Supplemental Table 6, available online at http://www.mcpiqojournal.org).

At the closest 12-month visit, 129/247 male-group and 139/250 female-group patients achieved a DAS28-CRP decrease ≥0.6. IPTW-weighted risks were 51.0% and 57.7%, respectively (RR 1.13, 95% CI, 0.94-1.36; *P*=.188). For a decrease ≥1.2, risks were 36.0% and 41.5% (RR 1.15; 0.90-1.48; *P*=.266) (Supplemental Table 7, available online at http://www.mcpiqojournal.org).

### Longitudinal ASDAS Trajectories by Rheumatologist Sex

The exploratory ASDAS-CRP model included 452 patients and 3244 observations (Figure 4). At 12 months, the estimated difference (women-men) in ASDAS-CRP was +0.143 (95% CI, −0.143 to 0.429; *P*=.327). Estimates at 24 and 36 months were similarly small and nonsignificant (−0.042; 95% CI, −0.334 to 0.251; *P*=.779); and +0.135; 95% CI, −0.127 to 0.397; *P*=.311) (Figure 3).

**Figure 4:**
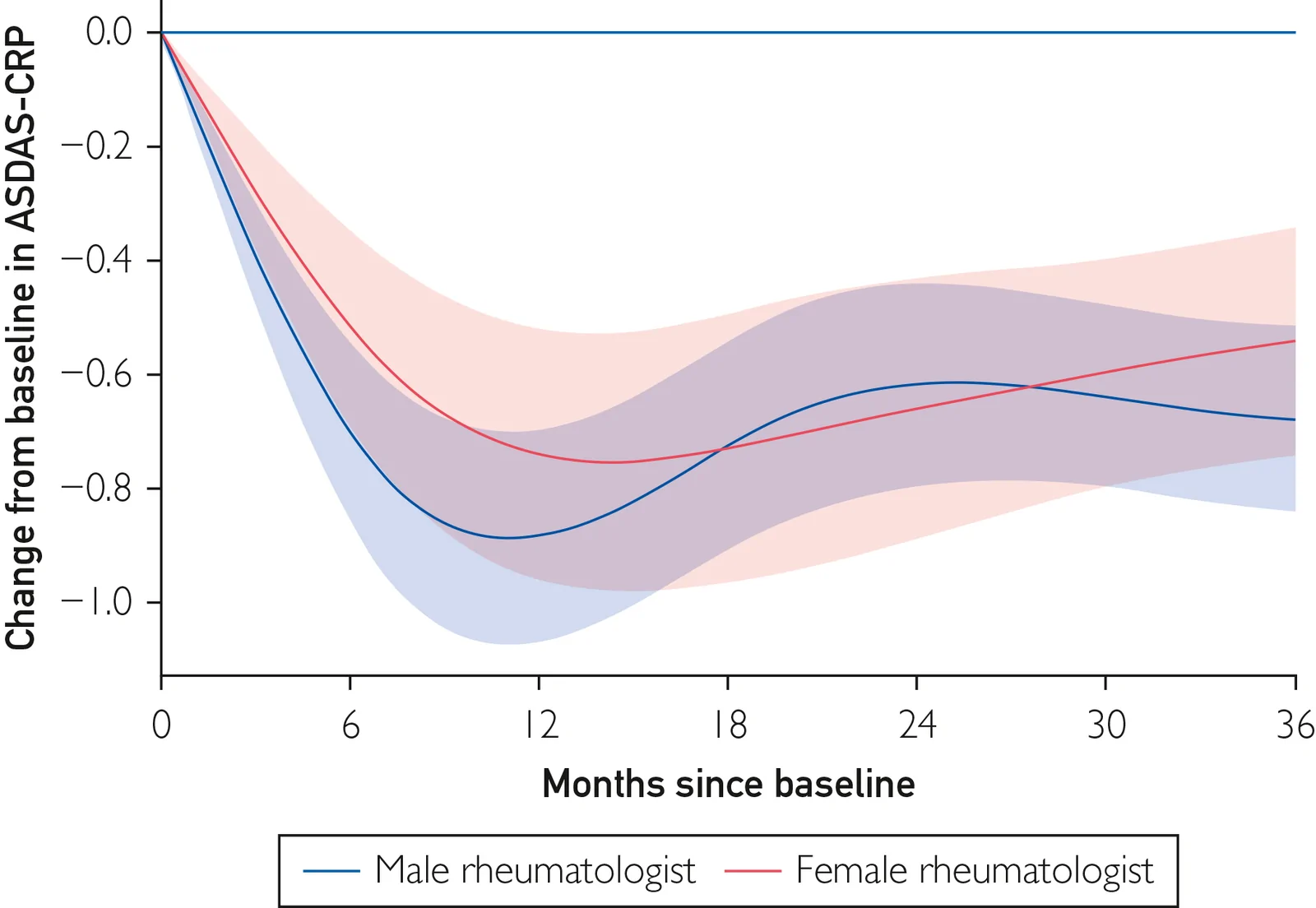
Predicted changes from baseline in ASDAS-CRP by baseline rheumatologist sex. Curves and 95% confidence bands derive from outcome-specific stabilized IPTW-weighted GEE models fitted in 50 imputed datasets and combined with Rubin's rules. ASDAS-CRP was exploratory and was not restricted to confirmed axial PsA. ASDAS-CRP, ankylosing spondylitis disease activity score based on C-reactive protein; GEE, generalized estimating equations; IPTW, inverse probability of treatment weighting; PsA, patients with psoriatic arthritis.

Among the ASDAS-CRP components, a women-minus-men difference in back pain was observed at 36 months (+1.084, 95% CI, 0.193 to 1.975; *P*=.017); all other prespecified back pain, peripheral joint pain/swelling, and morning stiffness contrasts included the null (Supplemental Figure 4, available online at http://www.mcpiqojournal.org). Visit-updated ASDAS-CRP differences were +0.166 (95% CI, −0.109 to 0.441), −0.056 (−0.303 to 0.192), and +0.081 (−0.146 to 0.308) at 12, 24, and 36 months, respectively. Stable-exposure, changed-exposure, and predominant-exposure analyses similarily showed no consistent association. At 12 months, weighted risk ratios were 0.98 (95% CI, 0.76 to 1.27; *P*=.895) for an ASDAS-CRP decrease ≥0.6 and 1.18 (0.81 to 1.72; *P*=.386) for a decrease ≥1.1.

### Subgroup Analyses: Patient Age and Sex, Physician Caseload

There was no clear evidence that 12-month associations differed by patient sex or age (Table 4). For DAS28-CRP, interaction p values were 0.209 for patient sex and 0.731 for age; for ASDAS-CRP, they were 0.771 and 0.156.

**Table 4:** Effect Modification by Patient Sex or Age Group (<65 vs ≥65 years) for the Primary and Secondary Outcomes at 12 Months

Outcome	Modifier	Group 0: difference (95% CI)	Group 1: difference (95% CI)	*P* interaction
DAS28-CRP	Patient sex	Male: −0.251 (−0.623 to 0.122)	Female: +0.111 (−0.319 to 0.541)	.21
DAS28-CRP	Age	<65: −0.092 (−0.396 to 0.213)	≥65: +0.064 (−0.761 to 0.888)	.73
ASDAS-CRP	Patient sex	Male: +0.102 (−0.292 to 0.497)	Female: +0.186 (−0.215 to 0.588)	.77
ASDAS-CRP	Age	<65: +0.223 (−0.087 to 0.533)	≥65: −0.394 (−1.173 to 0.386)	.16

Abbreviations: ASDAS-CRP, ankylosing spondylitis disease activity score based on C-reactive protein; CI, confidence interval; DAS28-CRP, 28-joint disease activity score based on C-reactive protein.

The median physician follow-up caseload was 38.5 patients, with 8 physicians per volume stratum. The interaction *P* value was .460. The low-volume 12-month responder comparison contained only 31 patients, including 3 managed by male rheumatologists, and was therefore too sparse for reliable effect modification inference **(**Supplemental Table 8, available online at http://www.mcpiqojournal.org).

### Effect of Sex of the Rheumatologists on b/tsDMARD Switching During Follow-Up

During follow-up, 230/410 patients in the male rheumatologist baseline group and 85/302 in the female rheumatologist group switched b/tsDMARD therapy at least once. Full-cohort weighted risks were 55.2% and 30.1%, respectively (RR 0.55; 95% CI, 0.43-0.70; *P*<.001). Weighted RRs were 0.52 (0.37-0.72) for exactly one switch and 0.60 (0.36-1.00) for multiple switches (Supplemental Table 9, available online at http://www.mcpiqojournal.org).

## Discussion

This large, real-world prevalent PsA cohort identified a time-specific association of physician sex with DAS28 improvement. The primary 12-month DAS28-CRP contrast was small and imprecise. A larger mean improvement was estimated at 24 months, whereas the 36-month estimate remained directionally similar. No corresponding association was found for ASDAS-CRP or for the prespecified DAS28-CRP responder thresholds.

The DAS28 difference at 24 months was not driven by inflammatory markers, as CRP trajectories were comparable between groups, but was mainly explained by reduced tender and swollen joint counts. In PsA, peripheral joint activity and axial involvement represent partially distinct disease domains, with CRP showing limited sensitivity to clinical disease activity, particularly in predominantly peripheral phenotypes.^15^

The lack of an association with ASDAS-CRP should be interpreted in light of important measurement limitations. In the present registry, axial involvement was not formally defined, and ASDAS components relied on symptom-based proxies that do not allow reliable discrimination between inflammatory and noninflammatory back pain.

The 24-month mean difference of −0.38 DAS28-CRP units was below the 0.6 threshold used for clinically important improvement at the individual level. Such findings should therefore be regarded as a modest population-average association that requires replication in different datasets. The direction of the 24-month estimate is compatible with observational literature relating physician characteristics to communication, care processes, and outcomes^4^, ^5^, ^6^, ^7^^,^^16^ and with our previous RA cohort.^14^ In rheumatology, evidence linking the sex of the treating physician to objective disease outcomes is limited and indirect, as most available studies were not designed to evaluate longitudinal disease activity and instead addressed patient-physician discordance or health care processes.^17^, ^18^, ^19^ Among US veterans with RA, PsA, or ankylosing spondylitis, factors associated with higher frequency of DMARD treatment included female diagnosing provider (HR 0.90; 95% CI, 0.85-0.96) and gender concordance between patient and provider (HR 0.91; 95% CI, 0.86-0.97).^20^ By explicitly modeling repeated measures over time and addressing baseline confounding using causal-inference-oriented methods, our study supports the hypothesis that physician-level factors may be associated with outcomes in PsA in ways not captured by earlier designs.

We observed no discernible effect modification by patient sex or age group. Estimates stratified by rheumatologist case volume likewise showed no clear heterogeneity; however, this analysis was limited by sparse and markedly unbalanced exposure groups and therefore cannot exclude a contribution of clinician workload. Overall, results were robust across multiple sensitivity analyses, particularly for the 24-month contrast. The visit-updated analysis yielded a similarly null estimate at 12 months, while negative between-group differences persisted at 24 and 36 months. This consistency supports the main pattern of findings.

Finally, switching of b/tsDMARD therapy occurred less frequently among patients managed by female rheumatologists. These findings were analyzed descriptively and should be interpreted as downstream consequences of clinical management rather than as confounders of the primary association.

### Study Limitations

Several limitations should be taken into account while providing insight for future research in this field. First, physician-patient allocation was not random and may have reflected clinic scheduling, physician availability, case mix, and previous care. The prevalent cohort design also entails left truncation: care before the first eligible registry visit was not observed. Second, only 16 physicians contributed at baseline, including 5 women. Physician sex may therefore proxy individual clinician, organizational, or scheduling characteristics, and inference based on a small number of physician-level exposure clusters may be optimistic. Third, baseline physician sex may misclassify subsequent exposure. Stable, predominant, and visit-updated analyses address different aspects of this problem but do not remove time-dependent confounding; stratification by post-baseline switching may itself select on disease course or service processes. Fourth, visit frequency and outcome availability were determined by routine care, creating potential informative follow-up. Fifth, joint counts were assessed by treating physicians who were not blinded to their own management, so observer-related differences cannot be separated fully from differences in inflammatory activity. Sixth, axial involvement was not formally defined, limiting ASDAS-CRP interpretation. Finally, the single-center setting limits generalizability, and sex was recorded as binary and should not be conflated with gendered behaviors.

## Conclusion

In this single-center prevalent PsA cohort, baseline-classified physician sex exposure was associated with modest differences in peripheral disease activity at later follow-up, but not with ASDAS-CRP. Concordant findings across stable and predominant-exposure analyses reduce concern about simple exposure misclassification; however, the observational design, small number of physicians, physician-level imbalance, and residual confounding preclude causal interpretation. Larger multicenter studies should examine specific, modifiable behaviors, including communication, uncertainty management, and shared decision-making, rather than physician sex itself.

## Potential Competing Interests

The authors report no competing interests.

## Ethics Statement

The study was approved by the Central-Eastern Veneto Area Territorial Ethics Committee with protocol number 6007/AO/24 in compliance with the principles of the Declaration of Helsinki as revised in 2000.

## Declaration of Generative AI and AI-Assisted Technologies in the Writing Process

During the preparation of this work the authors used ChatGPT in order to assist with implementation of predefined statistical analyses and preparation of figures. After using this tool, the authors reviewed and edited the content as needed and take full responsibility for the content of the publication.

## Supplemental Online Material

Supplementary_Material

STROBE Statement
