# Supplementary material for: Sex of the Treating Rheumatologist and Disease Activity Outcomes in Psoriatic Arthritis
Source: Mayo Clin Proc Innov Qual Outcomes. 2026 Sep 22;10(6):100761. doi: 10.1016/j.mayocpiqo.2026.100761 (PMC13676734; doi:10.1016/j.mayocpiqo.2026.100761)
Supplement: Supplementary file 1 [file mmc1.pdf]

Table S1. Baseline missingness and outcome-specific analytic samples

Variable	Missing overall, n	Missing overall, %	Missing male-rheumatologist group, n	Missing male group, %	Missing female-rheumatologist group, n	Missing female group, %
Disease duration	7	1,0	6	1,5	1	0,3
Comorbidities	173	24,3	97	23,7	76	25,2
DAS28-CRP	124	17,4	111	27,1	13	4,3
ASDAS-CRP	260	36,5	184	44,9	76	25,2
TJC28	124	17,4	111	27,1	13	4,3
SJC28	124	17,4	111	27,1	13	4,3
HAQ (source data)	584	82,0	311	75,9	273	90,4
Erosions	122	17,1	109	26,6	13	4,3
Radiographic progression	258	36,2	207	50,5	51	16,9
NSAID use	3	0,4	1	0,2	2	0,7

Table S2. Propensity-score and IPTW balance diagnostics

Variable	Observed n female	Observed n male	Unweighted |SMD| observed values	MI unweighted |SMD| completed values	MI-IPTW |SMD| completed values	Maximum MI-IPTW |SMD|	IPTW |SMD| observed values	IPTW |SMD| missingness indicator
Age, years	289	299	0,060	0,060	0,013	0,053	0,013	0,000
Female patient, n (%)	289	299	0,008	0,008	0,019	0,041	0,019	0,000
Disease duration, years	288	294	0,216	0,219	0,021	0,052	0,020	0,128
BMI, kg/m2	289	299	0,171	0,171	0,027	0,066	0,027	0,000
Obesity, n (%)	289	299	0,163	0,163	0,016	0,037	0,016	0,000
DAS28-CRP	289	299	0,232	0,232	0,029	0,054	0,029	0,000
ASDAS-CRP	213	115	0,099	0,133	0,028	0,078	0,164	0,548
SJC28	289	299	0,423	0,423	0,046	0,086	0,046	0,000
TJC28	289	299	0,356	0,356	0,023	0,071	0,023	0,000
PGA	289	299	0,095	0,095	0,032	0,075	0,032	0,000
HAQ	26	83	0,251	0,154	0,029	0,071	0,283	0,349
CRP, mg/L	289	299	0,100	0,100	0,033	0,094	0,033	0,000
Extra-articular manifestations, n (%)	289	299	0,058	0,058	0,016	0,044	0,016	0,000
Comorbidities, n (%)	218	219	0,248	0,191	0,015	0,063	0,033	0,056
Erosions present, n (%)	289	299	0,091	0,091	0,022	0,051	0,022	0,000
Radiographic progression in prior 12 months, n (%)	240	150	0,096	0,285	0,032	0,060	0,253	0,568
Glucocorticoid dose, mg/day	289	299	0,279	0,279	0,057	0,068	0,057	0,000
NSAID use, n (%)	287	298	0,202	0,202	0,017	0,051	0,019	0,034
Prior b/tsDMARD exposure, n (%)	289	299	0,177	0,177	0,011	0,033	0,011	0,000

Primary DAS28-CRP analytic sample n=588; summaries are means across 50 imputations unless stated otherwise

Trimmed stabilized IPTW across imputations: mean 0.99; mean SD 0.50; mean lower/upper truncation points 0.53 and 3.55; mean effective sample size 467.7.

The principal balance diagnostic is the absolute SMD in the completed covariate after IPTW. All mean completed-data SMDs were <0.10. The maximum column shows the largest SMD across the 50 completed datasets and is provided as a stability diagnostic, not as a separately pooled estimand.

Observed-value SMDs and missingness-indicator SMDs are reported separately to show differential ascertainment.

The additionally adjusted sensitivity model included disease duration, ASDAS-CRP, HAQ and radiographic progression, plus indicators that each was missing in the source data (Table S4).

Table S3. Sensitivity analyses for change in DAS28-CRP

Model	Month	Difference	95% CI low	95% CI high	p value	Interpretive role
Primary MI-IPTW; truncated; exchangeable; spline 4 df	12	-0,081	-0,367	0,205	0,58	Primary
Primary MI-IPTW; truncated; exchangeable; spline 4 df	24	-0,382	-0,621	-0,143	0,002	Primary
Primary MI-IPTW; truncated; exchangeable; spline 4 df	36	-0,262	-0,535	0,012	0,06	Primary
Primary + disease duration/ASDAS/HAQ/radiographic progression and missingness ir	12	-0,065	-0,346	0,216	0,65	Sensitivity
Primary + disease duration/ASDAS/HAQ/radiographic progression and missingness ir	24	-0,371	-0,608	-0,135	0,002	Sensitivity
Primary + disease duration/ASDAS/HAQ/radiographic progression and missingness ir	36	-0,249	-0,521	0,022	0,07	Sensitivity
Stabilized weights without truncation	12	-0,107	-0,416	0,203	0,50	Sensitivity
Stabilized weights without truncation	24	-0,383	-0,643	-0,124	0,004	Sensitivity
Stabilized weights without truncation	36	-0,286	-0,586	0,014	0,06	Sensitivity
AR(1) working correlation	12	-0,101	-0,408	0,206	0,52	Sensitivity
AR(1) working correlation	24	-0,372	-0,670	-0,074	0,01	Sensitivity
AR(1) working correlation	36	-0,319	-0,643	0,005	0,05	Sensitivity
Restricted cubic spline 3 df	12	-0,190	-0,439	0,059	0,13	Sensitivity
Restricted cubic spline 3 df	24	-0,351	-0,595	-0,107	0,005	Sensitivity
Restricted cubic spline 3 df	36	-0,371	-0,603	-0,139	0,002	Sensitivity
Restricted cubic spline 5 df	12	-0,077	-0,357	0,203	0,59	Sensitivity
Restricted cubic spline 5 df	24	-0,387	-0,647	-0,127	0,004	Sensitivity
Restricted cubic spline 5 df	36	-0,259	-0,536	0,017	0,07	Sensitivity
PS estimated in full n=712 then applied to DAS28 cohort	12	-0,043	-0,386	0,301	0,81	Sensitivity
PS estimated in full n=712 then applied to DAS28 cohort	24	-0,350	-0,624	-0,076	0,01	Sensitivity
PS estimated in full n=712 then applied to DAS28 cohort	36	-0,234	-0,555	0,087	0,15	Sensitivity
Outcome-specific PS excluding HAQ	12	-0,083	-0,366	0,200	0,57	Sensitivity
Outcome-specific PS excluding HAQ	24	-0,386	-0,622	-0,151	0,001	Sensitivity
Outcome-specific PS excluding HAQ	36	-0,267	-0,536	0,002	0,05	Sensitivity

Female-minus-male difference; negative values favor the female-rheumatologist group

The full-cohort PS sensitivity reproduces the strategy of estimating weights in n=712 before restricting to the DAS28-CRP sample; the outcome-specific PS is primary because balance was assessed and optimized in the actual analytic set.

Table S4. MI-IPTW GEE additionally adjusted for residual observed-data and missingness imbalance

Month	Primary difference	Primary 95% CI low	Primary 95% CI high	Adjusted difference	Adjusted 95% CI low	Adjusted 95% CI high	Adjusted p value
12	-0,081	-0,367	0,205	-0,065	-0,346	0,216	0,65
24	-0,382	-0,621	-0,143	-0,371	-0,608	-0,135	0,002
36	-0,262	-0,535	0,012	-0,249	-0,521	0,022	0,07

Primary DAS28-CRP analytic sample n=588; 4,416 observations

Adjustment covariates: imputed baseline disease duration, ASDAS-CRP, HAQ and radiographic progression, plus one indicator for whether each value was missing in the source data.

Model: Gaussian identity-link GEE, exchangeable working correlation, robust sandwich variance, restricted cubic spline for time (4 df), physician-sex-by-time interaction; Rubin pooling across 50 imputations.

Table S5. Visit-updated rheumatologist-sex sensitivity analysis

Outcome	Month	Predicted change - male rheumatologist at visit	Predicted change - female rheumatologist at visit	Female-male difference	95% CI low	95% CI high	p value
DAS28-CRP	12	-0,931	-0,981	-0,050	-0,326	0,227	0,72
DAS28-CRP	24	-0,551	-0,920	-0,369	-0,597	-0,142	0,001
DAS28-CRP	36	-0,617	-0,933	-0,317	-0,573	-0,061	0,02
ASDAS-CRP	12	-0,897	-0,731	0,166	-0,109	0,441	0,24
ASDAS-CRP	24	-0,608	-0,664	-0,056	-0,303	0,192	0,66
ASDAS-CRP	36	-0,653	-0,572	0,081	-0,146	0,308	0,49

Baseline outcome-specific IPTW retained; exposure updated at each recorded visit

Predictions are centered at time zero. Robust sandwich standard errors and Rubin pooling were used.

Table S6. Sensitivity analyses using alternative longitudinal exposure classifications

Outcome	Month	Exposure analysis	Patients	Male n	Female n	Difference	95% CI low	95% CI high	p value
DAS28-CRP	12	Baseline classification - all	497	247	250	-0,024	-0,321	0,272	0,87
DAS28-CRP	12	Stable physician sex	371	196	175	-0,001	-0,357	0,356	1,00
DAS28-CRP	12	Changed physician sex	126	51	75	-0,226	-0,717	0,265	0,37
DAS28-CRP	12	Predominant physician sex	497	233	264	-0,018	-0,313	0,277	0,91
DAS28-CRP	24	Baseline classification - all	406	198	208	-0,400	-0,706	-0,093	0,01
DAS28-CRP	24	Stable physician sex	292	148	144	-0,408	-0,768	-0,048	0,03
DAS28-CRP	24	Changed physician sex	114	50	64	-0,423	-1,010	0,163	0,16
DAS28-CRP	24	Predominant physician sex	406	183	223	-0,381	-0,686	-0,076	0,01
DAS28-CRP	36	Baseline classification - all	338	163	175	-0,525	-0,895	-0,154	0,01
DAS28-CRP	36	Stable physician sex	240	122	118	-0,491	-0,934	-0,047	0,03
DAS28-CRP	36	Changed physician sex	98	41	57	-0,626	-1,211	-0,041	0,04
DAS28-CRP	36	Predominant physician sex	338	153	185	-0,474	-0,839	-0,110	0,01
ASDAS-CRP	12	Baseline classification - all	387	191	196	0,126	-0,172	0,423	0,41
ASDAS-CRP	12	Stable physician sex	269	132	137	0,187	-0,143	0,516	0,27
ASDAS-CRP	12	Changed physician sex	118	59	59	-0,064	-0,659	0,532	0,83
ASDAS-CRP	12	Predominant physician sex	387	177	210	0,131	-0,161	0,423	0,38
ASDAS-CRP	24	Baseline classification - all	326	158	168	-0,077	-0,457	0,303	0,69
ASDAS-CRP	24	Stable physician sex	213	98	115	0,011	-0,376	0,398	0,95
ASDAS-CRP	24	Changed physician sex	113	60	53	-0,246	-1,039	0,547	0,54
ASDAS-CRP	24	Predominant physician sex	326	143	183	-0,111	-0,479	0,257	0,55
ASDAS-CRP	36	Baseline classification - all	268	126	142	-0,008	-0,292	0,275	0,95
ASDAS-CRP	36	Stable physician sex	170	74	96	0,175	-0,181	0,531	0,34
ASDAS-CRP	36	Changed physician sex	98	52	46	-0,301	-0,752	0,149	0,19
ASDAS-CRP	36	Predominant physician sex	268	117	151	0,081	-0,197	0,360	0,57

Closest observed visit within +/-6 months; patient-level weighted mean differences pooled across imputations

Table S7. Twelve-month responder analyses

Outcome	Response threshold	Patients	Male events / n	Female events / n	Weighted risk male, %	Weighted risk female, %	Risk ratio	95% CI low	95% CI high	p value
DAS28-CRP	Decrease >=0.6	497	129/247	139/250	51,0	57,7	1,132	0,941	1,361	0,19
DAS28-CRP	Decrease >=1.2	497	94/247	99/250	36,0	41,5	1,153	0,897	1,482	0,27
ASDAS-CRP	Decrease >=0.6	387	96/191	104/196	53,9	53,0	0,983	0,759	1,272	0,89
ASDAS-CRP	Decrease >=1.1	387	61/191	73/196	34,6	40,8	1,181	0,811	1,720	0,39

Closest observed visit within +/-6 months; outcome-specific MI-IPTW

Risk ratios compare the female- with the male-rheumatologist baseline group. Confidence intervals include uncertainty within and between imputations.

Table S8. Characteristics of baseline rheumatologists

Anonymized ID	Sex	Birth year	Age in 2019	Patients at baseline	Distinct patients over follow-up	Recorded visits	Study-period volume group
R1	Female	1958	61	266	312	2071	High
R2	Female	1958	61	15	27	108	Low
R3	Female	1959	60	11	27	180	Low
R4	Female	1953	66	6	9	40	Low
R5	Female	1962	57	4	30	95	Low
R6	Male	1957	62	102	153	1218	High
R7	Male	1951	68	83	83	151	High
R8	Male	1966	53	73	123	276	High
R9	Male	1955	64	58	168	704	High
R10	Male	1952	67	39	47	137	High
R11	Male	1955	64	33	86	266	High
R12	Male	1974	45	18	61	118	High
R13	Male	1954	65	1	2	2	Low
R14	Male	1981	38	1	1	1	Low
R15	Male	1978	41	1	1	2	Low
R16	Male	1979	40	1	1	2	Low

Table S9. Changes in b/tsDMARD agent during follow-up

Switching outcome	Male events / 410	Female events / 302	Weighted risk male, %	Weighted risk female, %	Risk ratio	95% CI low	95% CI high	p value	Interpretation
Any switch (>=1)	230	85	55,2	30,1	0,545	0,425	0,699	<0,001	Post-baseline descriptive outcome
Exactly one switch	158	58	37,7	19,6	0,518	0,374	0,718	<0,001	Post-baseline descriptive outcome
Multiple switches (>=2)	72	27	17,5	10,5	0,602	0,362	1,001	0,05	Post-baseline descriptive outcome

Full eligible cohort n=712; full-cohort MI-IPTW

A switch was defined as a change between consecutive non-missing recorded b/tsDMARD agent codes. Class changes alone were not required.

Supplementary Figure S1. Propensity-score overlap and stabilized-weight distributions

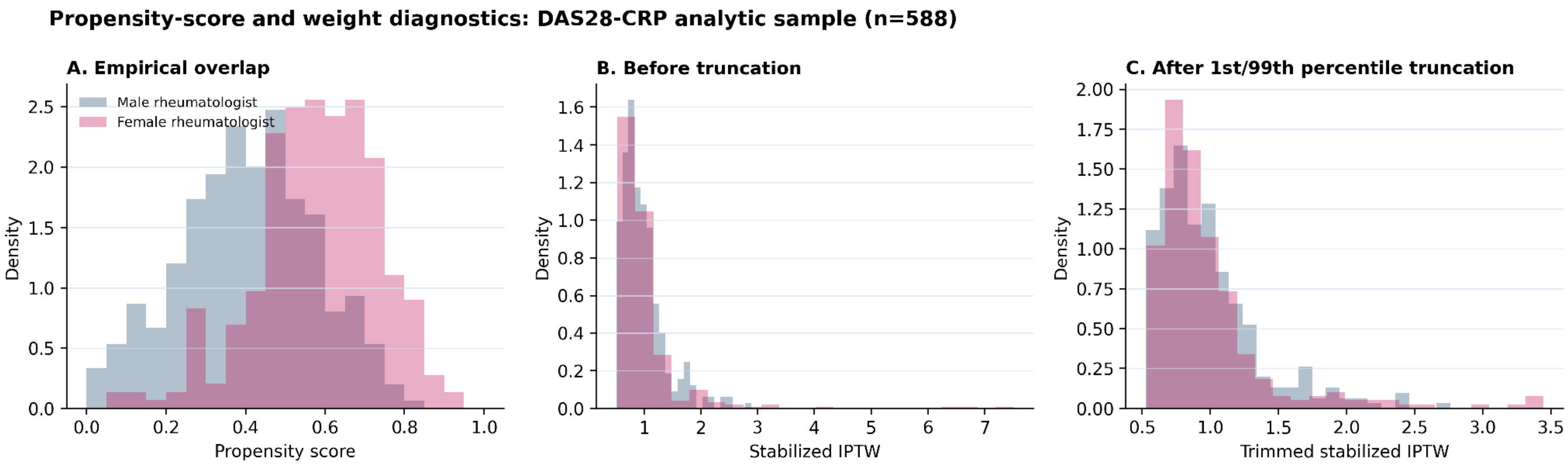

Distributions are based on each patient's mean propensity score or weight across 50 completed datasets in the primary DAS28-CRP analytic sample. Panels show overlap and the influence of 1st/99th percentile truncation. Patient-level values are not tabulated.

Supplementary Figure S2. Forest plot of DAS28-CRP sensitivity analyses

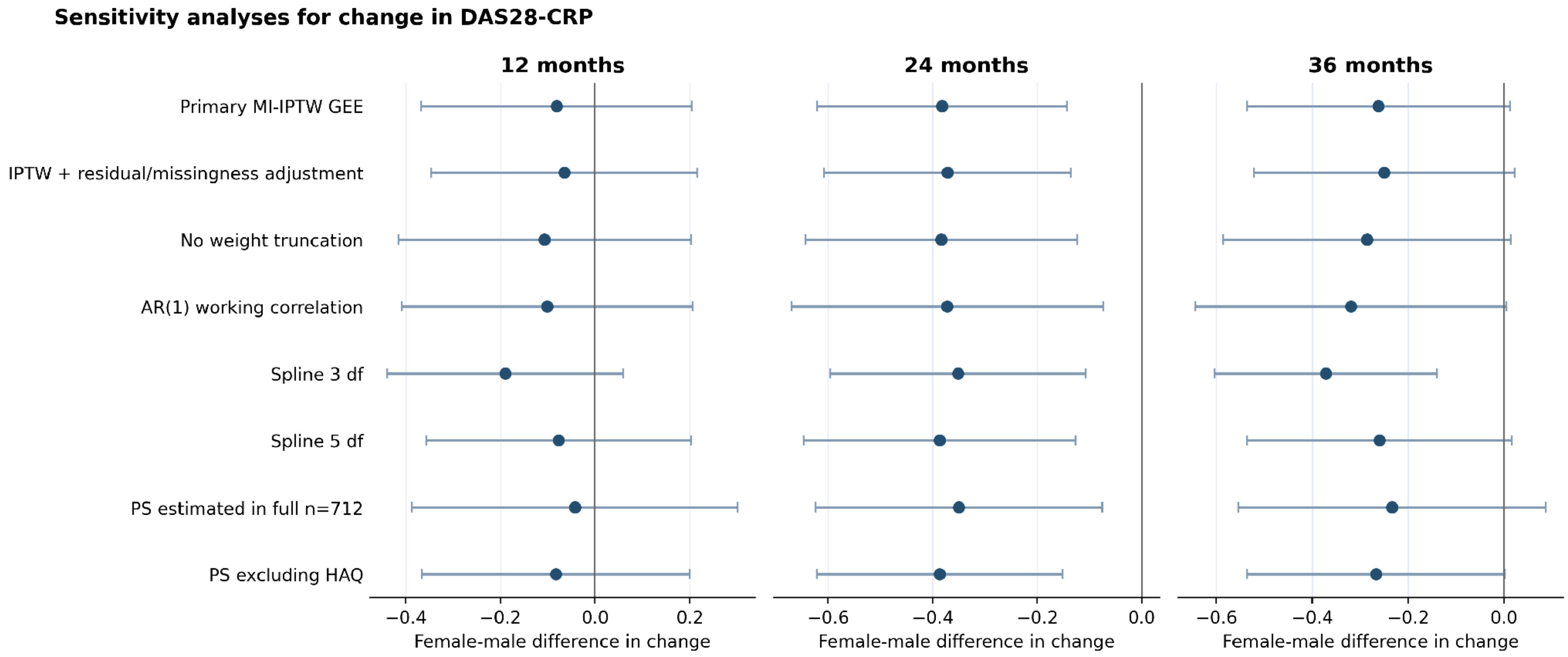

Female-minus-male differences in change from baseline at 12, 24 and 36 months. Points are estimates and bars are 95% confidence intervals. The 24-month direction was consistent across specifications; 36-month precision depended partly on spline specification.

Supplementary Figure S3. Visit-updated rheumatologist-sex sensitivity analysis

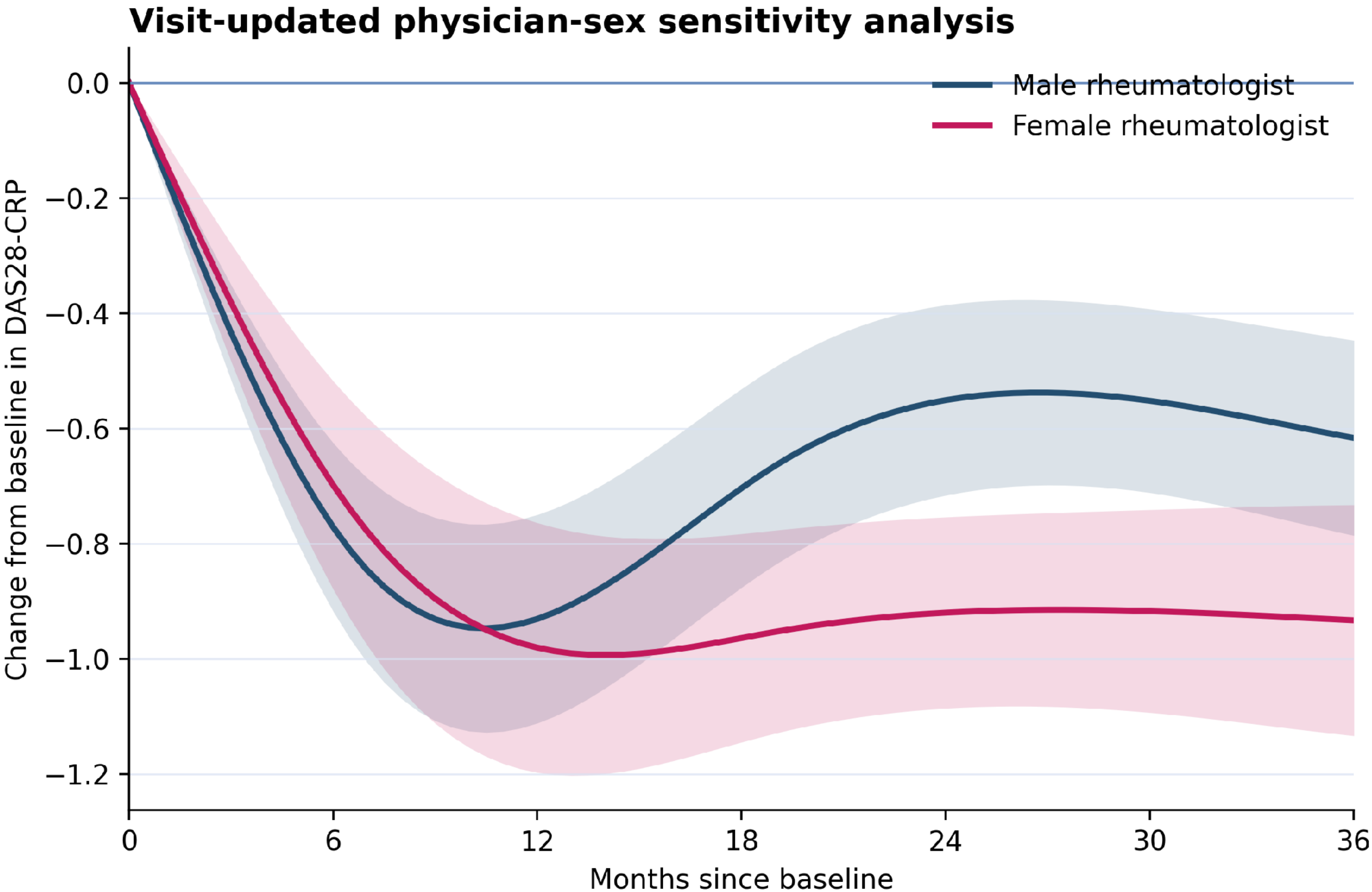

Supplementary Figure S4. Time-dependent trajectories of ASDAS-CRP components

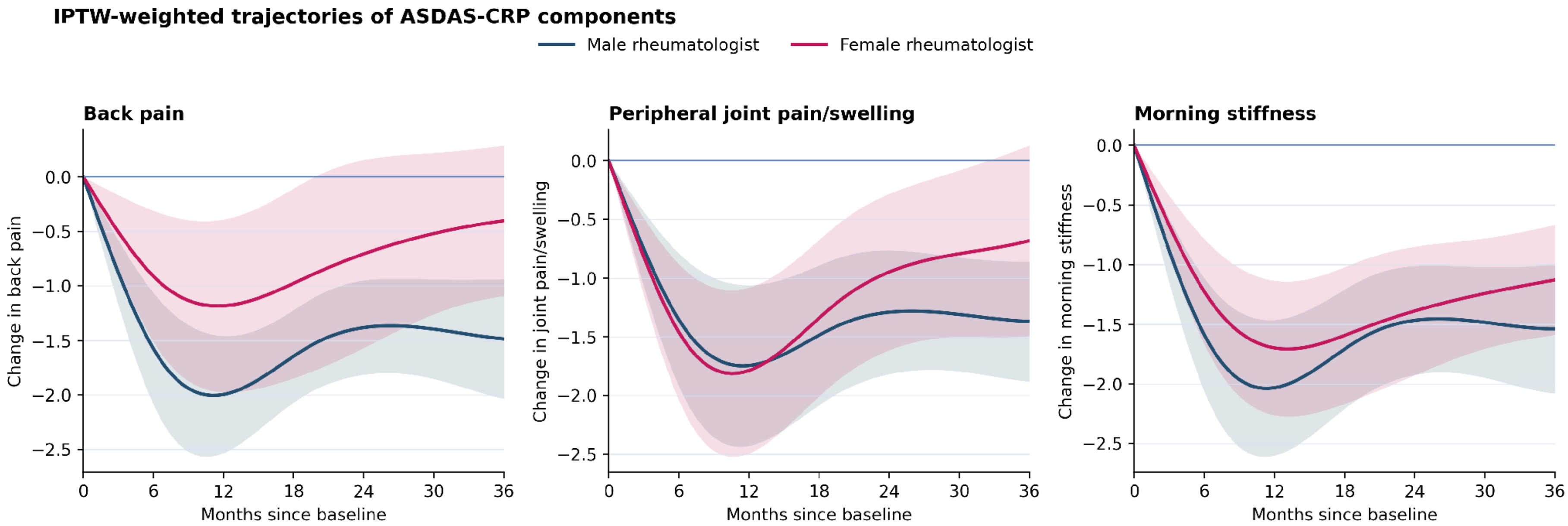

MI-IPTW GEE trajectories for back pain, peripheral joint pain/swelling and morning stiffness by baseline rheumatologist sex. Shaded bands show 95% confidence intervals. Component analyses are exploratory and were not multiplicity-adjusted.
